# Imaging atelectrauma in Ventilator-Induced Lung Injury using 4D X-ray microscopy

**DOI:** 10.1038/s41598-020-77300-x

**Published:** 2021-02-19

**Authors:** Luca Fardin, Ludovic Broche, Goran Lovric, Alberto Mittone, Olivier Stephanov, Anders Larsson, Alberto Bravin, Sam Bayat

**Affiliations:** 1 European Synchrotron Radiation Facility, Grenoble, France; 2 Hedenstierna Laboratory, Department of Surgical Sciences, Uppsala University, Uppsala, Sweden; 3 Center for Biomedical Imaging, EPFL, Lausanne, Switzerland; 4 Swiss Light Source, Paul Scherrer Institute, Villigen, Switzerland; 5 CELLS - ALBA Synchrotron Light Source, Barcelona, Spain; 6 Department of Pathology, Grenoble University Hospital, Grenoble, France; 7 Synchrotron Radiation for Biomedicine Laboratory (STROBE, INSERM UA7), Grenoble, France; 8 Department of Pulmonology and Physiology, Grenoble University Hospital, Bd. Du Maquis du Grésivaudan, 38700 La Tronche, France

**Keywords:** Respiration, Respiratory distress syndrome, Imaging techniques, X-rays

## Abstract

Mechanical ventilation can damage the lungs, a condition called Ventilator-Induced Lung Injury (VILI). However, the mechanisms leading to VILI at the microscopic scale remain poorly understood. Here we investigated the within-tidal dynamics of cyclic recruitment/derecruitment (R/D) using synchrotron radiation phase-contrast imaging (PCI), and the relation between R/D and cell infiltration, in a model of Acute Respiratory Distress Syndrome in 6 anaesthetized and mechanically ventilated New-Zealand White rabbits. Dynamic PCI was performed at 22.6 µm voxel size, under protective mechanical ventilation [tidal volume: 6 ml/kg; positive end-expiratory pressure (PEEP): 5 cmH_2_O]. Videos and quantitative maps of within-tidal R/D showed that injury propagated outwards from non-aerated regions towards adjacent regions where cyclic R/D was present. R/D of peripheral airspaces was both pressure and time-dependent, occurring throughout the respiratory cycle with significant scatter of opening/closing pressures. There was a significant association between R/D and regional lung cellular infiltration (*p* = 0.04) suggesting that tidal R/D of the lung parenchyma may contribute to regional lung inflammation or capillary-alveolar barrier dysfunction and to the progression of lung injury. PEEP may not fully mitigate this phenomenon even at high levels. Ventilation strategies utilizing the time-dependence of R/D may be helpful in reducing R/D and associated injury.

## Introduction

Mechanical ventilation is an essential means of life support for patients with acute respiratory failure. It is necessary in the treatment of patients with acute respiratory distress syndrome (ARDS)^1^, a pathologic condition characterized by severe lung inflammation, diffuse alveolar damage and infiltration, increased microvascular permeability edema, surfactant dysfunction and widespread patchy atelectasis^2^. The overall mortality of ARDS remains high: between 35 and 45%^3^, in part because despite its vital role in improving oxygenation, mechanical ventilation can damage the lungs, a condition referred to as Ventilator-Induced Lung Injury (VILI). Identifying ventilation strategies that provide adequate gas exchange while minimizing the progression of lung injury remains an urgent and open question. However, the mechanisms leading to VILI at the microscopic scale remain poorly understood, and better knowledge of these mechanisms is of fundamental importance for addressing this question.

It is generally agreed that the main mechanism of worsening pre-existing lung injury under assisted ventilation is the mechanical strain imposed on the lung tissue, which can have multiple origins^4^. Overdistension due to high regional tidal volumes can induce mechanical injury to the lung, through exaggerated stress and strain. Evidence to support this hypothesis is mainly based on clinical studies showing the improvement in the mortality of ARDS patients by reducing tidal volume^5^. The link between tidal stretch of the lung tissue and regional inflammatory responses has also been demonstrated in experimental studies^6^.

Cyclic reopening (recruitment) of lung regions that become re-collapsed (derecruited) upon expiration, a phenomenon thought to induce “atelectrauma”, can also contribute to the expansion of lung injury, due to high mechanical stresses normal to the epithelial surface of the airways as they reopen^7^. High stress or strain is also produced at the interface between adjacent collapsed and aerated alveoli, especially within poorly aerated parenchyma leading to increased local stress or ‘stress concentration’^8^.

Positive end-expiratory pressure (PEEP) is usually applied in ARDS to improve oxygenation by preventing end-expiratory lung collapse. It should theoretically prevent atelectrauma and reduce the inhomogeneities responsible for stress concentration. Three large trials, investigating the effects of PEEP on mortality of ARDS patients in the intensive care setting have failed however in showing an improvement in overall survival^9–11^, and a decrease in mortality was observed only in a sub-category of severe ARDS patients^12^. The importance of atelectrauma in the development of VILI is therefore still unclear.

Here, we investigated the pressure–time dependency of atelectrauma during protective ventilation and the regional relation between atelectrauma and cell infiltration in an early-stage rabbit model of VILI. We used 4D (3D + time) in vivo phase-contrast microscopy with a 22.6 × 22.6 × 22.6 μm^3^ voxel size and monochromatic X-rays produced with a synchrotron radiation (SR) source. Synchrotron sources deliver radiation that is orders of magnitude more intense than conventional X-ray sources, which is essential for high temporal and spatial resolution imaging. 4D X-ray phase-contrast microscopy combined with phase retrieval algorithms^13^, allows improving the contrast-to-noise ratio of poor X-ray attenuating media, such as the lung tissue^14^. A novel image acquisition method was developed in order to resolve the lung parenchymal motion induced by both respiration and cardiac activity, a major challenge at this high spatial resolution.

## Results

### Lung injury

The PaO_2_/F_IO2_ ratio was 461 ± 52; 78 ± 27 (*p* < 0.001) and 107 ± 119 (*p* < 0.001) at baseline, after injury induction and at the end of the experiment, respectively (Supplementary Table S1). An increase in the average PaO_2_/F_IO2_ at the end of the experiment was due to one animal, which met the conditions for VILI after injury induction but recovered during the following imaging session.

**Figure 1:**
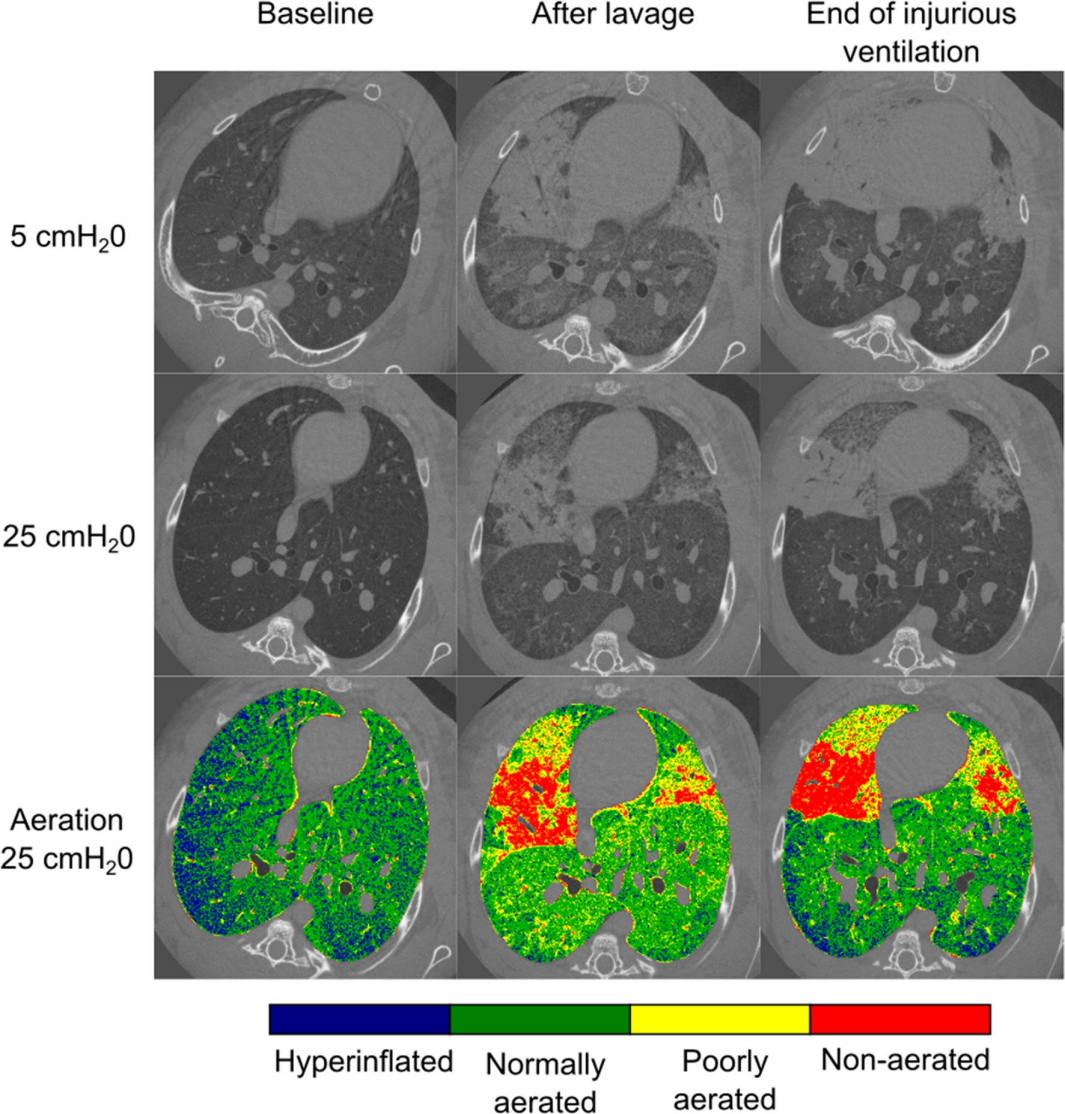
CT section of Rabbit-1 acquired in apnea at baseline, immediately after the lavage, and at the end of injurious ventilation, at two different tracheal pressures (5 cmH_2_O and 25 cmH_2_O). Note that the experiments were performed with the animals in upright position. Aeration levels are shown for each time step at 25 cmH_2_O. Non-aerated tissue (red) corresponds to the interval (HU ≥ − 100); poorly aerated tissue (yellow) to (− 500 ≤ HU < − 100); normally aerated tissue (green) to (− 900 ≤ HU < − 500 HU) and hyperinflated tissue (blue) to (HU < − 900 HU). Atelectatic regions develop around lung units which became atelectatic immediately after the lavage.

In Fig. 1, static CT images acquired upon expiration (5 cmH_2_O) and inspiration (25 cmH_2_O) are shown in a representative animal. These images were acquired at baseline and the end of the two phases of our lung injury model: lavage-induced surfactant depletion followed by injurious ventilation. A map of lung aeration was computed based on X-ray attenuation at each pressure (5, 25 cmH_2_O) and experimental condition: baseline; lavage; injurious ventilation. Immediately after lavage-induced surfactant depletion, patchy areas of high X-ray attenuation due to poorly-aerated and non-aerated lung regions appeared. At the end of the injurious ventilation, repeated static images showed distinct changes compared to post-lavage.

**Figure 2:**
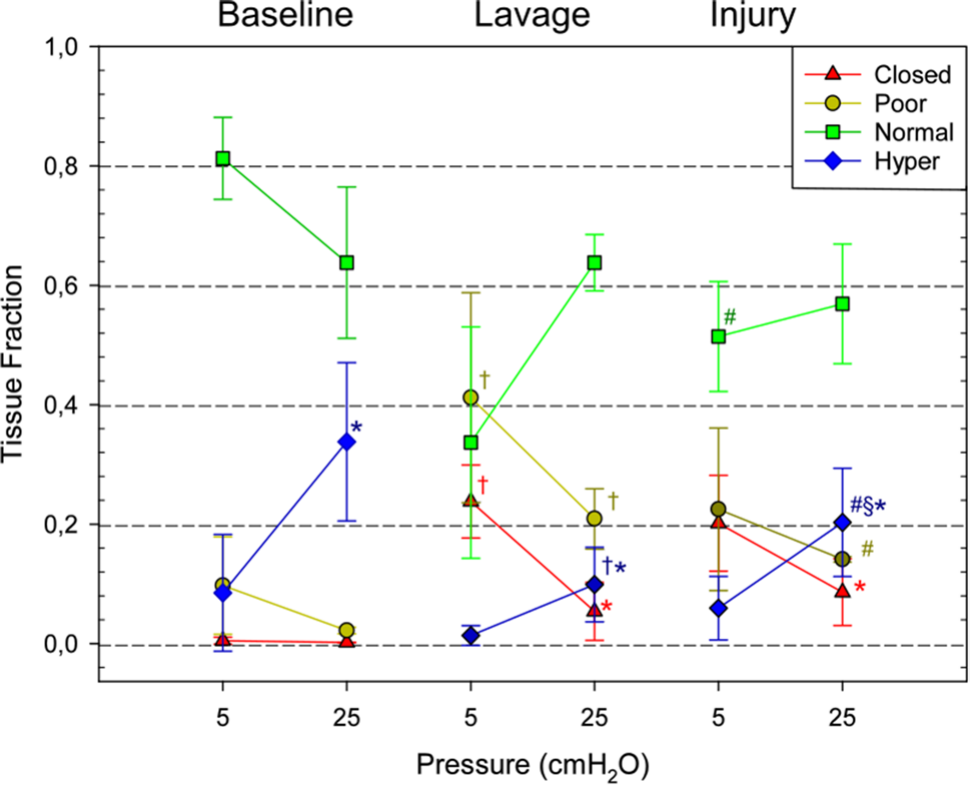
Average fraction of closed, poorly aerated, normally aerated and hyperinflated lung regions as a function of time and pressure at baseline, after whole lung lavage, and at the end of the injurious ventilation (n = 4). *p < 0.05 versus PEEP5 within experimental condition; †*p* < 0.05 versus baseline within PEEP (lavage); #*p* < 0.05 versus baseline within PEEP (injury); §*p* < 0.05 vs lavage within PEEP.

The lung tissue was divided into non-aerated, poorly-aerated, normally-aerated and hyperinflated regions. The average fractions of each lung aeration category are shown in Fig. 2 as a function of experimental condition and pressure. At baseline, increasing airway pressure reduced poor aeration, and as expected, increased hyperinflation, in line with our previous observations in this model^15^. After lavage, there was a significant increase in poorly-aerated and non-aerated regions, which were recruited at 25 cmH_2_O. Following injurious ventilation, the area of poorly-aerated regions decreased, while the area of normally aerated zones increased in proportion, suggesting that this may have been due to active epithelial fluid resorption. On the other hand, closed regions persisted or worsened and tended to be less recruited, suggesting remaining fluid-filled or “sticky” atelectatic regions. The latter are apparent in particular upon inspiration. Finally, hyper-inflated regions at 25 cmH_2_O significantly increased in the injured condition, which may have been due to a reduced compliance and less recruitment of poorly- and non-aerated regions, or the so-called “baby lung” effect.

**Figure 3:**
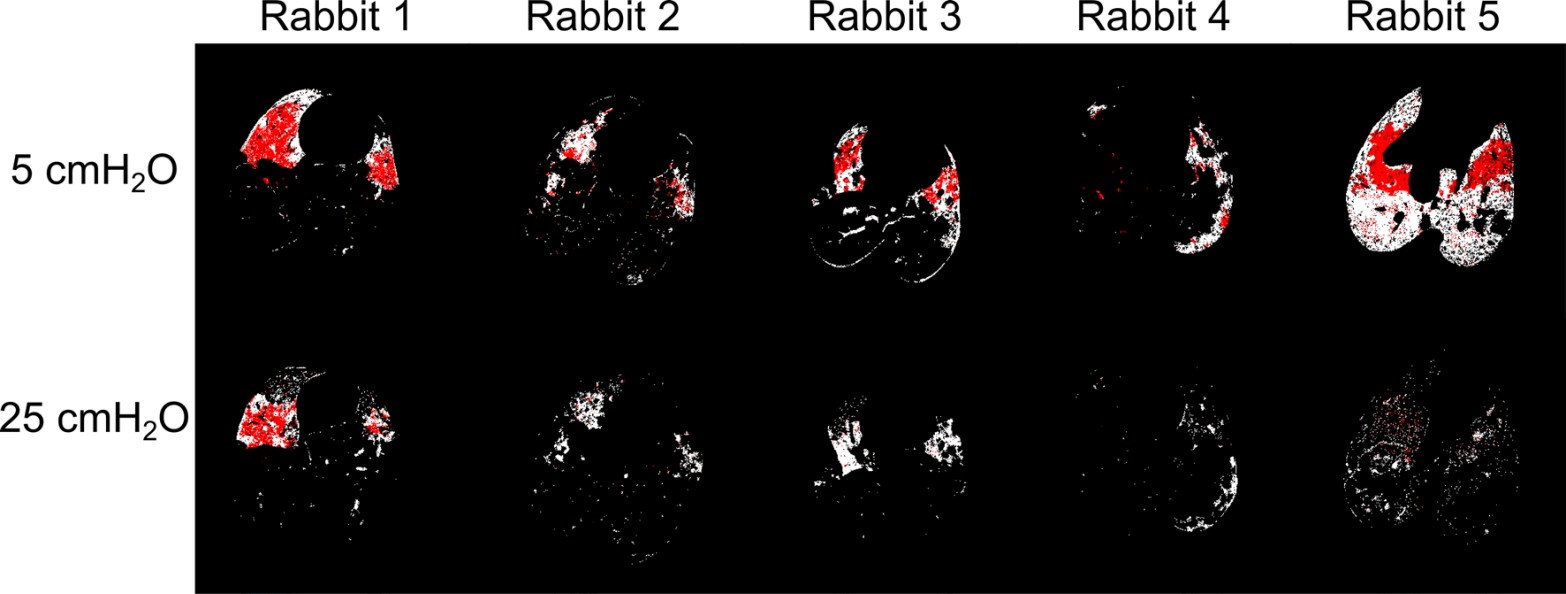
Maps of non-aerated lung regions after injurious ventilation. The maps refer to the static CT images acquired during expiration (5 cmH_2_O, upper panel) and inspiration (25 cmH_2_O, lower panel). White: non aerated regions after injurious ventilation; Red: non aerated regions after injurious ventilation which were already present after saline lavage. A spatial co-localization between non-aerated regions before and after injurious ventilation is visible at a pressure of 5 cmH_2_O. Recruitment of non-aerated regions upon inspiration is visible after saline lavage (lower panel).

A spatial analysis of the pattern of injury progression during injurious ventilation is shown in Fig. 3. This figure represents a map of the non-aerated regions after injurious ventilation, for all the rabbits and at the two pressures. The airspaces, which were non-aerated already after saline lavage, are highlighted in red. The co-localization of atelectatic regions between saline lavage and injury at low pressure, suggests that injury developed peripheral to lung regions which were atelectatic immediately after the saline lavage. The recruitability of these regions, visible at high pressure, suggests atelectrauma as a mechanism of injury progression.

### Dynamics of atelectrauma

**Figure 4:**
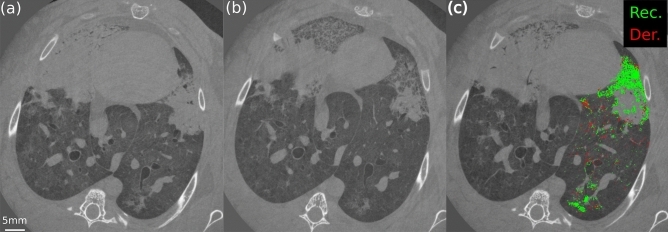
In vivo dynamic CT of Rabbit-1 at minimum pressure at the beginning of inspiration (5 cmH_2_O) (**a**) and at maximum pressure at the end of the inspiration (25 cmH_2_O ) (**b)**. In (**c**) a 2D projection of the recruitment (Rec., green) and derecruitment (Der., red) as identified by the algorithm in the left lung, superimposed to one CT slice as a reference. (**c**) represents the time instant, during the respiratory cycle, where maximum recruitment was observed.

A 3D animation (4D reconstruction) of the whole respiratory cycle was obtained in all animals after injurious ventilation. The animation of a 2D slice in one representative animal is included in the Supplemental Digital Content (Supplementary Video S1). A dynamic map of within-tidal recruitment (green) and derecruitment (red) of airspaces was obtained for each 4D image reconstruction, by cross-correlation of corresponding voxels at consecutive time frames. As an example, an animation showing a dynamic recruitment/derecruitment (R/D) map is provided in the Supplemental Digital Content (Supplementary Video S2), superimposed to the corresponding animation of the respiratory cycle. Single 2D frames from the reconstructed 4D images, respectively at end-expiration (5 cmH_2_O) and end-inspiration (25 cmH_2_O), are shown in Fig. 4a,b in one representative animal (Rabbit-1). These images represent a dynamically derecruited and a recruited state, respectively. A snapshot of a R/D dynamic map at end-inspiration is shown in Fig. 4c. It represents a 2D projection of the R/D events identified by the algorithm, at this time point. Either one or both lungs were analyzed for each animal: the right lung had to be discarded in Rabbit-1 and Rabbit-5 due to the presence of the diaphragm and of the stomach in the field of view, and in Rabbit-4 due to motion artefacts caused by a mechanical instability of the tomographic setup.

**Figure 5:**
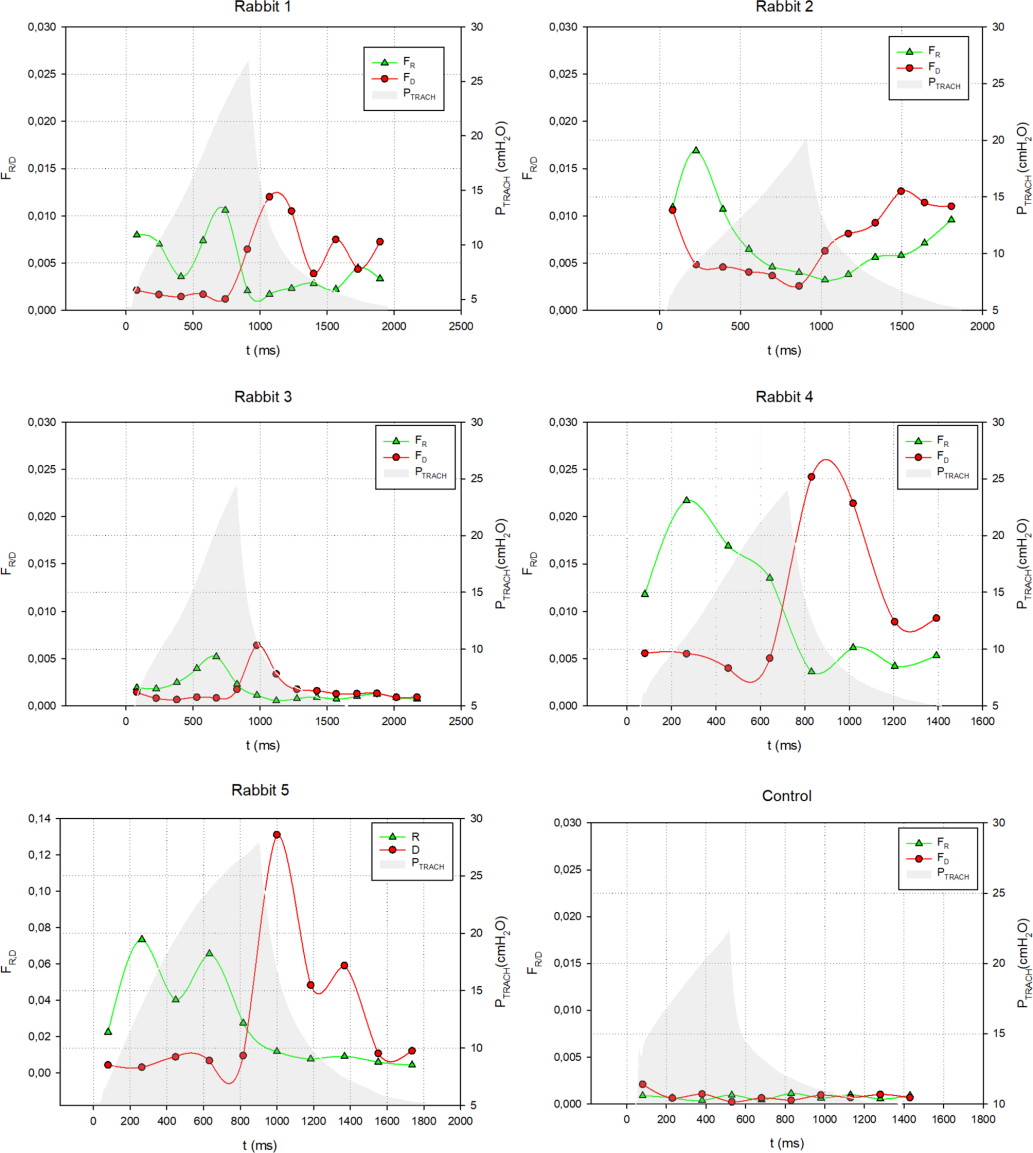
Graphs representing the recruitment (F_R_) and derecruitment (F_D_) fraction as a function of time for all the rabbits. The pressure–time curve is shown as a reference. Rabbit-5, having a different injury model, is represented as last graph, with a different scale for the recruitment and derecruitment fraction.

In Fig. 5, the R/D fractions are shown as a function of time for each 4D reconstruction, superimposed to the corresponding tracheal pressure–time curves. There was a notable disparity in airway opening and closing times and pressures within and in between rabbits. Recruitment occurred both at low pressures at the beginning of inspiration (Rabbit-2,-4,-5) and at high pressures at the end of inspiration (Rabbit-1,-3,-5). Similarly derecruitment is identified both at high pressures at the beginning of inspiration (Rabbit-1,-3,-4,-5) and at low pressures at the end of expiration (Rabbit-1,-2,-5). In general, a coexistence of recruitment and derecruitment is identified all along the respiratory curve, in these dynamic images. The control rabbit showed only small fluctuations in both the recruitment and derecruitment curves with values close to the baseline of the injured animals.

### Comparison of atelectrauma and histological analysis

**Figure 6:**
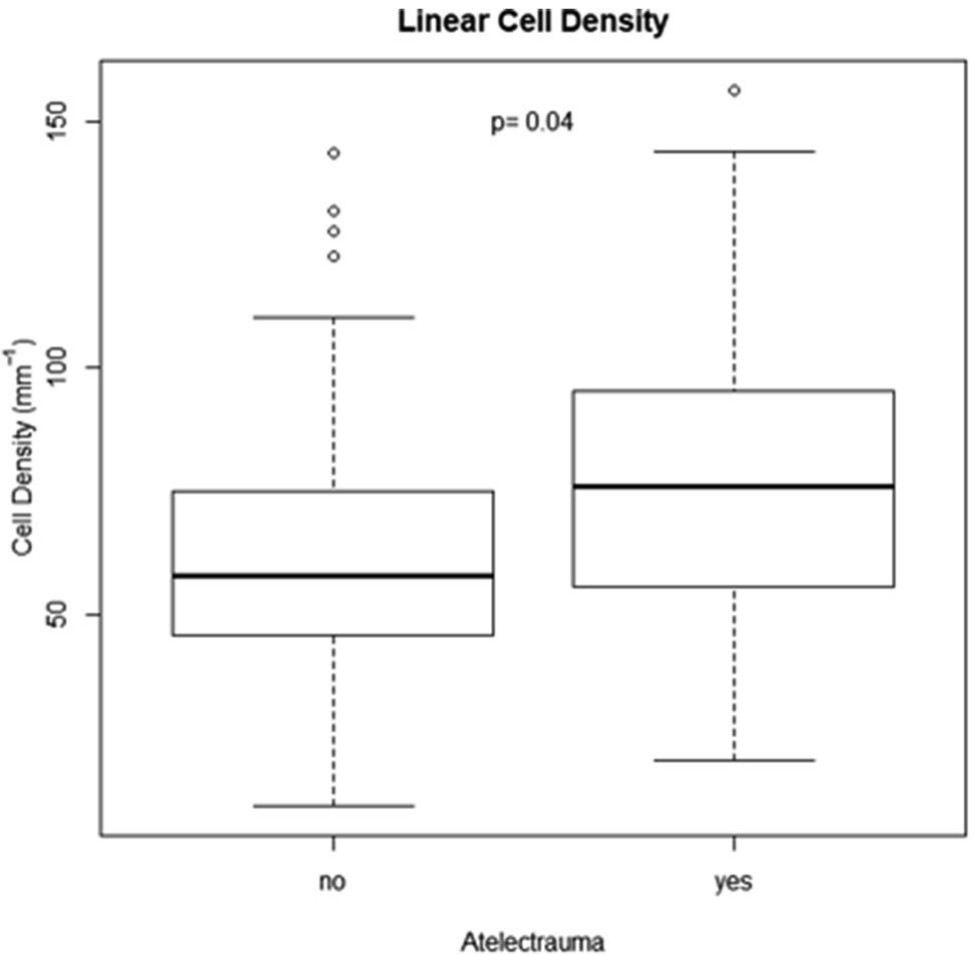
Cell counts per unit tissue length, comparing ROIs with (yes) and without (no) atelectrauma. Cell counts were greater in atelectatic regions with *p* = 0.04 following the Wilcoxon rank-sum test.

There was a significant relation between the R/D visible in the in vivo CT images and the tissue damage identified by pathology (*p* = 0.007). Moreover, we observed a significant difference in the linear cell densities within versus outside of lung regions with atelectrauma (*p* = 0.04). A higher mean value was observed in regions where atelectrauma occurred, showing neutrophil and macrophage infiltrations (Fig. 6).

## Discussion

The goal of this study was to characterize both the spatial distribution and the dynamic behaviour of atelectrauma using in vivo 4D phase-contrast microscopy at subacinar spatial resolution, in order to better understand whether and how this phenomenon contributes to the development of lung tissue damage and the extension of lung parenchymal injury. Our main findings were that: (1) in the presence of a mechanically inhomogeneous lung due to surfactant depletion, injury propagated outwards from non-aerated lung regions to adjacent lung submitted to atelectrauma; (2) recruitment and derecruitment of peripheral airspaces is not only a pressure-dependent, but also a dynamic, time-dependent phenomenon occurring throughout the respiratory cycle; (3) there was a co-localization of atelectrauma and lung cellular infiltration, a hallmark of inflammation and capillary-alveolar barrier dysfunction.

The pattern of injury progression in our model of VILI (Figs. 1 and 3) is in agreement with previous observations of VILI complicating pre-existing lung injury. Cereda et al. found that injury progressed concentrically in mechanically-ventilated rats with HCl-induced acute lung injury, meaning that secondary VILI originates adjacent to the primary lesions, due to the local diffuse intermingling of aerated and collapsed tissue^16^. They suggested that local discontinuities in aeration in poorly-aerated regions concentric to non-aerated zones of “sticky” atelectasis or edema, may lead to stress concentration in these zones upon inspiration. Using K-edge subtraction imaging of Xe gas, we previously found faster Xe wash-in in such poorly-aerated regions in a similar model, which may have been caused by reciprocal dilation of ventilated airspaces near collapsed or fluid-filled alveoli^15^. The significance of these findings is that alveolar collapse and edema result in a heterogeneous pattern of inflation which leads to concentration of stress within the aerated alveoli adjacent to closed ones, a phenomenon referred to as *stress risers*^17^. This is further demonstrated by the induction of atelectasis by bronchial blockage in rats with otherwise normal lungs, where 3 h of mechanical ventilation induced significant alveolar damage, edema and neutrophilic infiltration in the peri-atelectatic regions^18^. Together, these data suggest a significant role of stress concentration in regions adjacent to non-recruiting alveolar collapse in the propagation of lung injury and VILI.

Our findings further extend these observations by revealing the pattern and dynamics of atelectrauma at a sub-acinar spatial resolution. Data on the dynamics of atelectrauma or tidal R/D are scarce, and to our best knowledge, this is the first study of these phenomena at such spatial and temporal resolution in intact lung. Spatial analysis of our images demonstrated that regions adjacent to the initial injury are highly subjected to cyclic R/D (Fig. 3). This phenomenon leads to a large dynamic alveolar strain, which is thought to be a primary mechanism of VILI^19–22^. Moreover, we found significant scattering in the pressure and time at which airspaces open and close, both within and in-between animals (Fig. 5), while the 4D CT images were acquired under protective ventilation conditions, with low tidal volumes and a PEEP of 5 cmH_2_O.

Derecruitment of airspaces was observed at almost all pressures, including at values close to the higher end of the driving pressure, implying that even moderate to high PEEP levels may not completely prevent atelectrauma. This finding extends previous studies showing that recruitment in early lung injury occurs along the entire inspiratory limb of the PV curve^23–31^. Using dynamic CT at lower spatial resolution in a whole-lung lavage model of lung injury in pig^25^, David et al. showed that the volume of cyclic lung R/D was independent of the level of PEEP. Also, we previously found that cyclic R/D was reduced but still occurred despite PEEP levels up to 12 cmH_2_O^32^. In this study, using a resolution higher than in previous work^33^, we show that recruitment of collapsed or edematous airspaces does not lead to fully open airspaces, but rather a fine mosaic of micro-unaerated lung regions (Fig. 1). This suggests that although PEEP may increase the overall lung aeration, new poorly-aerated inhomogeneous lung regions may be created^15^, exposing these zones to tidal R/D and consequent stress concentration^33^. Beyond the adverse hemodynamic effects of high PEEP, this mechanism may be involved in the lack of improved survival in randomized clinical trials comparing higher versus lower PEEP levels in patients with ARDS^10,11,34^ with the exception of a subgroup of more severe lung injury^12^.

An important finding of this study is the coexistence of both recruitment and derecruitment during the whole respiratory cycle. Although counter-intuitive, one explanation of this finding is the strong time-dependence of cyclic R/D, due to the viscoelastic nature of alveolar opening and collapse^35^. In other words, recruitable alveoli open once a critical opening pressure is exceeded, however with a time lag, which in turn depends on the tissue micromechanical properties and airway liquid surface tension^35^. Moreover, the temporal pattern of recruitment is not continuous but rather a succession of “avalanches”^36^. Importantly, mechanical interdependence of neighbouring alveoli dynamically modifies the critical airway opening and closing pressures of the airspaces^32^. Because of the large scattering of the time constants and pressures of opening and closure in injured lung, some airspaces may recruit while airway pressures are in the descendent phase and vice versa. Evidence for the time-dependence of lung recruitment has previously been demonstrated in lavage injured pig lungs where the time-constant of recruitment ranged from 8 to 16.8 s^37^. This observation is also in line with our previous findings using static phase-contrast CT as well as in silico modelling of R/D phenomena^32,38,39^.

There is little data in the literature linking the regional dynamic mechanical behaviour of the lung with local cellular infiltration, reflecting tissue injury through inflammation or capillary-alveolar barrier dysfunction^40–42^. Indeed, quantification of inflammation is usually performed without data on spatial distribution, in the bronchoalveolar lavage fluid by counting neutrophils, measuring cytokine levels, or myeloperoxidase activity^43^. In our study, the regional histological analysis showed that, atelectrauma at the subacinar length scale is associated with increased inflammatory cell infiltration (Fig. 6), a hallmark of inflammation^44^ and capillary-alveolar barrier dysfunction, in this model of VILI. Recently, Yen et al. compared regional cytokine expression and specific tidal volume using 4D-CT in endotoxin-induced lung injury in mice^45^. Although there was no significant association between regional cytokine expression and specific tidal volume, IL-6 expression was increased in regions with a smaller end-expiratory volume, which could indicate a role of poor regional aeration. However, R/D or regional parenchymal strain were not assessed in that study. Four-D microscopy, coupled to histological analysis, did however allow spatial correlation between lung micromechanics and a biological hallmark of VILI in the present study.

### Study limitations

This study had several limitations. We were able to study only one PEEP level. We can therefore not make definitive conclusions on how PEEP would modify the progression of injury due to atelectrauma. However, the within-tidal dynamics of atelectrauma did allow us to assess opening and closing of airspaces as a function of pressure as well as time, therefore our hypothesis is based on data. A second limitation is that the animal was imaged in upright position, due to the horizontal geometry of the radiation beam, a constraint intrinsic to our imaging setup. This means that there is no gravitational gradient within our images, and that there is no superimposed pressure on the dorsal lung regions. We know from previous studies that this position slightly but significantly improves the respiratory mechanics in rabbit^46^. Image analysis was based on structural correlation through image registration. As any image registration-based approach is prone to artefact, particularly at the interface of larger vessels and airways, as well as at the outer boundaries of the lung. Successive filtering and morphological operations were performed to remove artefacts originating from the registration procedure. A one-to-one pixel correspondence between opening and closing airspaces within the images and histologic slides is not feasible. However, care was taken to identify anatomical landmarks such as airway or vascular bifurcations in order to ensure the best possible match between ROI’s.

## Conclusions

Using in vivo 4D phase-contrast microscopy at sub-acinar spatial resolution, we found that in the presence of a mechanically inhomogeneous lung due to surfactant depletion, injury propagates outwards from non-aerated lung regions to adjacent lung submitted to atelectrauma. Recruitment and derecruitment of peripheral airspaces is not only a pressure-dependent, but also a time-dependent phenomenon occurring throughout the respiratory cycle. Moreover, there was an association between R/D and regional lung cellular infiltration, a hallmark of inflammation and capillary-alveolar membrane dysfunction. Our data suggest that even higher PEEP levels may not fully mitigate R/D phenomena in the inured lung. These data also suggest that ventilation strategies aiming at reducing the time during which respiratory pressure is lowered during expiration may help reducing R/D phenomena.

## Methods

### Experimental setup

The experiments were performed at the biomedical beamline (ID17) of the European Synchrotron Radiation Facility (ESRF), Grenoble, France. A description of the beamline can be found in Elleaume et al.^47^. Briefly, the X ray produced by one of the two multipole wigglers were monochromatized by a double bent Laue Si(111) monochromator^47,48^, placed ~ 140 m from the source, selecting an energy of 52 keV. A sample to detector distance of approximately 1.5 m was set to take advantage of propagation-based phase contrast imaging^13^. X-rays were converted to visible light with a 50 µm thick Gadox scintillator and detected by a PCO Edge 5.5 camera (PCO AG, Kelheim, Germany)^49^ coupled to optics giving a measured effective pixel size of approximately 22.6 µm. The field of view was reduced to 2560 × 200 pixels, corresponding to 57.9 × 4.5 mm^2^ (horizontal × vertical, respectively). Data were transferred and saved in the ESRF central storage server, through a 10 Gb/s network. The transfer and saving speed determined an upper limit to the frame rate achievable, equal in this experiment to 100 fps with the aforementioned field of view.

### Animal preparation

The care of animals and the experimental procedures were in accordance with the Directive 2010/63/EU of the European Parliament on the protection of animals used for scientific purposes and were approved by the Ethical committee #113 (Ethax) under the authorization number 2015091517388915. The study was performed on six male New Zealand White Rabbits (Wt: 2.71 ± 0.15 kg); five with induced VILI and one control. Anesthesia was induced by intramuscular injection of Ketamine (10 to 15 mg/kg) and Xylazine (3 to 6 mg/kg). A tracheostomy was performed with insertion of a tightly secured 3.5-F plastic catheter (Portex, Smiths Medical, Minneapolis, Minnesota, USA). A 24-G catheter (Venflon, BD, Franklin Lakes, New Jersey, USA) was placed on the central ear artery, to monitor blood pressure. After surgery the animal was immobilized in the vertical position in a custom-made plastic holder and placed in the experimental hutch for imaging. Anesthesia was maintained during the duration of the experiment with IV infusion of Ketamine (80 mg/kg/h) (Ketamine 1000, Virbac, France) and Xylazine (8 mg/kg/h) (Paxman, Virbach, France). After verifying adequate depth of anesthesia, muscle relaxation was induced by Atracurium infusion (0.6 mg/kg/h) to avoid motion and suppress spontaneous breathing. Mechanical ventilation was administered using a custom-made system as described previously^50,51^ (Supplementary Fig. S1).

The electrocardiogram (ECG), tracheal pressure (P_TRACH_), tracheal flow and arterial pressure were continuously recorded using a Powerlab 16/35 data acquisition device (DAQ, Adinstruments, Dunedin, New Zealand). The signals were sampled at 10 kHz. Blood gas analysis was performed using the i-STAT System analyzer (Abbot Point of Care Inc., Princeton, NJ USA).

### 4D microscopy acquisition protocol

The motion of the lung parenchyma is induced by the combined action of heart contraction and mechanical ventilation, two asynchronous sources of motion. In order to avoid motion artefact in the reconstructed images, mechanical ventilation and the heartbeat were synchronized (Supplementary Fig. S2), by triggering the respiratory cycle with the R peaks of the ECG. The R waves of the ECG, acquired by the Powerlab, were detected using the Labchart software (ADInstruments, Dunedin, New Zealand), with a thresholding algorithm. When an R wave was detected a square wave was generated, with user defined duration and duty cycle. The square wave was transmitted via a TTL signal generated by the Powerlab to the electromagnetic valve in the expiratory branch of the mechanical ventilator, controlling its status (closed; open) with a preset inspiration/expiration time. The ECG polling was disabled for the whole duration of the square wave. When the square wave ended, a new polling on the ECG signal began. The following respiratory cycle was started when the next R peak was detected. The average period of the cardiac cycle was on the order of 300–350 ms, which was too short to obtain a sufficient ventilation: the duration of the square wave included therefore 4 to 5 cardiac cycles. When this synchronization protocol was applied, the mechanical ventilator was adapted to allow for the control of the expiratory valve with the Powerlab. The ventilator was switched back to its standard configuration after each synchronized scan.

When the heart rate is stable, it can be assumed that the motion of the lung parenchyma is periodic: the phase of the motion is fully determined by the ECG through two parameters (Supplementary Fig. S2): the interval from the jth R peak (Φ_j_ = Δt_j_) and the j index itself, denoting which of the n-heart pulsations included in the respiratory cycle is considered. Under these assumptions, a retrospective gated acquisition protocol was adopted: image projections were acquired at a constant frame rate and retrospectively sorted to reconstruct one CT for each phase of the parenchymal motion.

A complete CT was acquired during a single slow 2π rotation of the sample, using the half acquisition technique. In parallel beam geometry (which is almost perfectly satisfied by using synchrotron radiation: horizontal divergence: < 0.5 mrad), it allows to almost double the field of view of the camera for large samples by moving the axis of rotation towards the edge of the detector^52^.

### Experimental protocol

At baseline, the animals were ventilated with a protective ventilation strategy, with: V_T_ = 5–6 ml/kg, FiO_2_ = 0.5; PEEP = 5 cmH_2_O. VILI was induced through surfactant depletion by 4 whole-lung saline lavages (40 mL each) followed by injurious ventilation, with: FiO_2_ = 1; PEEP = 0 cmH_2_O and a peak inspiratory pressure (P_MAX_) = 30 cmH_2_O. The development of injury was monitored at regular intervals of 30 min, through blood gas analysis performed on PEEP 5 cmH_2_O, and through static chest tomography performed in apnea at PEEP 5 cmH_2_O and 25 cmH_2_O, respectively. Static chest tomography was performed at the same pressures at baseline and immediately after the saline lavage, as a reference. The injurious ventilation was stopped when the PaO_2_/F_i_O_2_ ratio was below 200 mmHg (moderate-acute lung injury^2^) and mechanical ventilation was switched back to protective ventilation. One 4D (3D + time) microscopy scan of the injured lung was acquired. A novel acquisition protocol, based on the synchronization between heartbeat and mechanical ventilation, was specifically designed to minimize motion artefacts as described in the previous section.

During the 4D microscopy acquisition, mechanical ventilation was synchronized with the ECG, with the following parameters: PEEP = 5 cmH_2_O; F_i_O_2_ = 1; respiratory rate = 30 to 40 breaths per minute; inspiratory/expiratory ratio = 1:2; driving pressure = 15 to 25 cmH_2_O. For the acquisition of the 4D microscopy the integration time was set to 3 ms and the frame rate to 90 fps. The speed of rotation was set to cover 1000 respiratory cycles in a 2π rotation, determining a total scan duration of approximately 30 min, with 147,500 projections acquired.

In one animal (Rabbit 5) 1.2 mL of iodine (400 mg ml^−1^) was added to the 40 mL saline solution for the lavage, to better visualize the distribution of fluid within the airspaces.

After imaging, each animal was euthanized by barbiturate overdose (IV injection of pentobarbital sodium 220 mg/kg); the heart and lungs were removed *en-bloc* and fixed by instillation of Formaldehyde 4% through the tracheal cannula at a hydrostatic pressure of 20 cmH_2_O. The extraction began on average 4 h after the beginning of the injurious ventilation, 30 min after the euthanasia. The fixed samples were sliced axially and embedded in paraffin. Lung tissue sections (3 µm) were prepared on glass slides and stained with hematoxylin, eosin and saffron (HES). The slides were scanned under light microscopy at × 10 magnification using a LEICA Aperio AT2 microscope (Leica microsystem, Wetzlar, Germany).

### 4D microscopy reconstruction

The phase of the lung parenchymal motion, at which each projection was acquired, was determined prior to tomographic reconstruction. Thanks to the synchronization between the heartbeat and the mechanical ventilator, the phase was fully determined by the ECG. The ECG acquired during the scan was analyzed with a Matlab script (version 2017a, www.mathworks.com), to identify the QRS complexes based on the approach described in^53^: this approach is based on a band pass filtering in the wavelet domain. Once the QRS complexes were identified, a feedback on the detection was obtained computing the time interval between consecutive complexes, corresponding to the duration of the heartbeats, expected to be constant or slowly varying. The window of the band pass filter in the wavelet domain was empirically adjusted in case of wrong QRS identifications.

Given a specific delay $\overline{{\Delta {\text{t}}_{{\text{j}}} }}$ from the j-th QRS complex of the periodic respiratory cycle, which labels the phase of the parenchymal motion, all the projections whose acquisition started in a time interval centered around $\overline{{\Delta {\text{t}}_{{\text{j}}} }}$ and with amplitude T equal to the integration time, were used for the reconstruction of the corresponding phase:$\phi_{{\text{j}}} = \overline{{\Delta {\text{t}}_{{\text{j}}} }}$. Tomographic reconstruction was performed with a Filtered Back Projection algorithm using the software PyHST2^54^ after the application of the Paganin’s single material phase retrieval algorithm^55^. Since the ECG signal is not perfectly periodic, the projections were not equally spaced. The angle associated to each projection was computed knowing the speed of the rotation and the time elapsed since the beginning of the acquisition.

For each dynamic scan, 10 to 15 phases were reconstructed. Since the selection of the projections is based on the ECG analysis, the phases corresponding to the QRS complexes of each heart cycle were reconstructed, together with the phases at 150 ms time distance. Since the duration of the heart cycle was of approximately 300–350 ms, the phases chosen were almost equally spaced in time. If the heart cycle was longer than 375 ms, a third phase was reconstructed per heart period, positioned at 300 ms from the previous QRS complex. The choice of having one image approximately every 150 ms was a compromise between time resolution and computational time.

### Detection of atelectrauma

4D microscopy was used to study and quantify atelectrauma dynamically during a whole respiratory cycle. To this end, volumes corresponding to consecutive timeframes were realigned using elastic image registration and the density of corresponding regions was compared, to identify opening and closure of airspaces. Prior to elastic registration volumes were rescaled by a factor of two, to reduce the computation time, and denoised by the application of an anisotropic diffusion algorithm^56^ implemented in the Simple ITK library (Insight Segmentation and Registration Toolkit, https://itk.org).

Image registration was performed using the Simple Elastix library^57,58^. An initial affine transformation was estimated to compensate for the vertical translation of the lungs during ventilation, followed by a 3D B-Spline model. The cost function utilized was the sum of square differences of the volumes, with the bending energy regularization term described in Rueckert et al.^59^, coupled to a gradient descent optimizer. A multiresolution approach was utilized, with scaling factors 8, 4 and 2, achieving a minimum voxel size of 80^3^ μm^3^. The spacing of the grid, on which the B-Spline approximation was defined, was adapted to the different resolutions: 1.6 mm for the scaling factor 8 and 4 and 0.8 mm for the highest resolution. A Gaussian smoothing, with standard deviation equal to one half of the scaling factor, was applied when downsampling. The computation time was 1 h on a local desktop machine (2 CPUs: Intel Xeon @ 2.3 GHz ⨯ 18, 512 GB of RAM).

Once two consecutive time frames were realigned, a threshold on the gray levels was applied. The threshold was empirically defined based on the histogram of the gray levels, to divide the two main distributions: the one of the aerated voxels and the one of blood vessels and soft tissue. Since a given threshold corresponds to a precise value of air fraction in the voxel (estimated as 45% in this study), the threshold was considered constant along the respiratory cycle and in between rabbits. Opening occurred when the status of the voxels changed from non-aerated to aerated, and vice-versa. Since the registration did not have a one-voxel precision, a morphological opening with a 3D kernel of radius 1 was applied to remove detections due to residual misalignments of airways and blood vessels.

Atelectrauma was quantified by defining the recruitment and derecruitment fractions F_R_ and F_D_, respectively equal to the volume detected as opening and closing between the time frames i and i + 1 $\left( {V_{R,D}^{i } } \right)$, normalized to the total lung volume in the field of view at time frame i $\left( {V^{i} } \right)$:

$${\text{F}}_{R,D}^{i} = \frac{{V_{R,D}^{i} }}{{V^{i} }}$$

The normalization was adopted to take into account the inflation and deflation during mechanical ventilation and for inter-rabbit comparison.

### Assessment of injury development

To assess how injury developed in our model, the static tomographic scans acquired at PEEP 5 cmH_2_0 and 25 cmH_2_0 during injurious ventilation were reconstructed using Filtered Back Projection and after applying the Paganin’s single material phase retrieval algorithm. The analysis was focused on three conditions: baseline, saline lavage and end of injurious ventilation. The gray levels were converted to Hounsfield Units (HU) assuming a linear relationship, which was guaranteed by the linearity of the detector^49^ and the monochromaticity of the beam. The gray level and HU of blood and air were utilized as reference points for the conversion. The linear attenuation coefficient for the blood at 50 keV was obtained from the NIST database and was used to compute the nominal HU value of blood at this energy. The lung tissue in each tomographic reconstruction was segmented into 4 components: Closed (HU > − 100), Poorly aerated (− 500 < HU < − 100), Normally Aerated (− 900 < HU < − 500) and Hyper-inflated (HU < − 900)^60^. Since the intervals found in literature refer to a lower spatial resolution, the volumes were downscaled by a factor 10 in the reconstruction plane before conversion to HU. A sub-acinar spatial resolution could lead to a higher air content inside each voxel and to the resulting overestimation of hyperinflation. The fraction corresponding to each of the four categories was computed, by dividing by the total lung tissue volume in the field of view at each time and pressure. The pressure–time dependence of the tissue fractions was considered an indicator of lung injury development and was evaluated with a repeated measurements two-way ANOVA, considering time and pressure as factors. The analysis was performed for each tissue category separately. The two-way interaction between the factors was performed with a paired t-test using the Bonferroni correction for the *p*-value. The statistical tests were performed with the software R (version 3.6.3).

### Assessment of alveolar damage and inflammation

**Figure 7:**
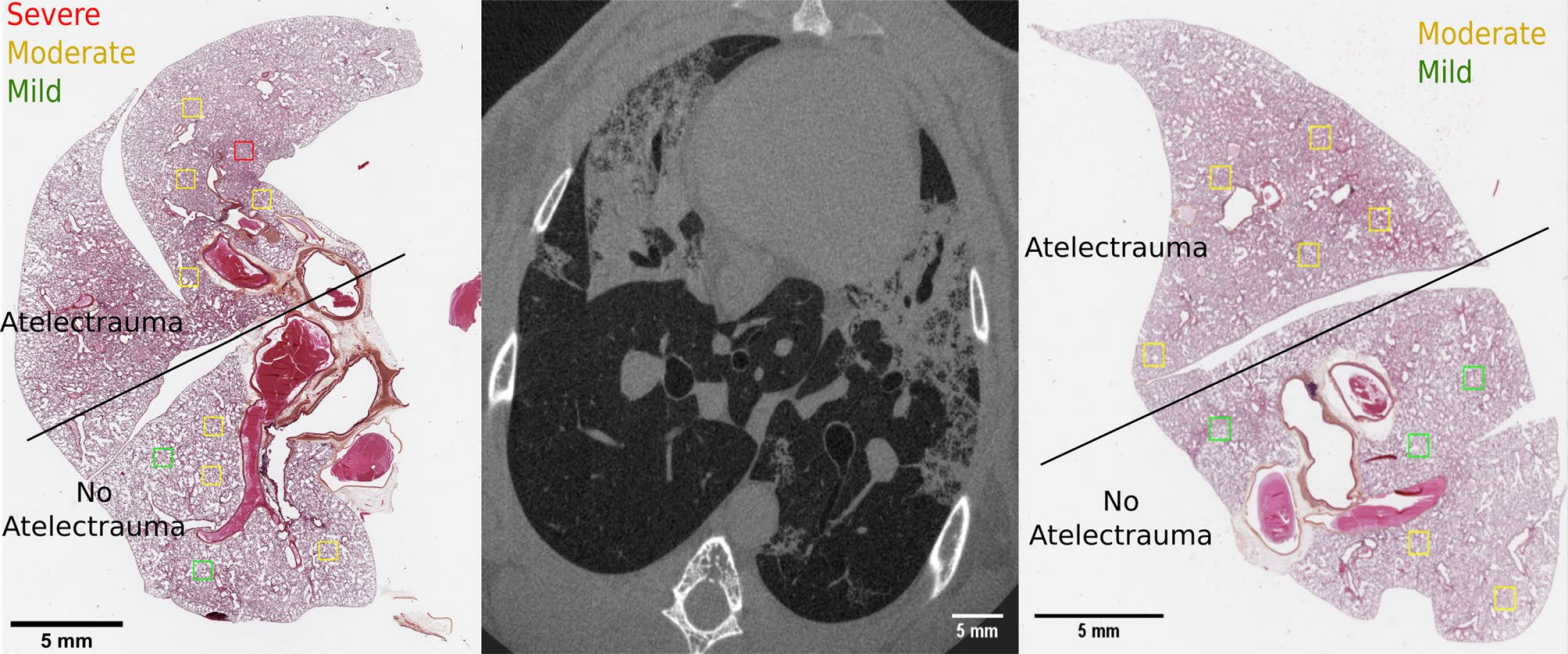
HES staining of the lung tissue and corresponding in vivo dynamic 3D microscopy for Rabbit 3. On the HES images, the regions of interest chosen for the qualitative evaluation are shown, using a color coding representing the score given by the pathologist. The regions of interest were chosen in areas showing R/D in the CT and in areas not showing it, in order to compare the spatial distribution of R/D and injury.

Correlation between alveolar damage and atelectrauma was assessed by histological analysis, based on a qualitative description of the injury (Fig. 7). Regional lung cellular infiltration was further quantified by automated cell counting. For each lung, one histologic section was chosen, yielding a total of ten samples. For each sample ten regions of interest (ROI) of 800 $\times$ 800 μm^2^ were chosen as representative of two classes: (i) atelectrauma visible in the 4D microscopy; (ii) normally aerated during the whole respiratory cycle. A one-to-one correspondence between CT and histology couldn’t be established, due to the morphological changes occurring during the sample preparation; CT and histology couldn’t therefore be realigned. Blood vessels and airways were used as landmarks to manually identify corresponding regions with the best accuracy achievable.

The qualitative assessment of the injury in the 100 regions of interest was performed by a pathologist blinded to the sample condition, with a score from 1 to 4, corresponding to: none; mild; moderate and severe injury, similar to Tsuchida et al.^61^. The score was based on neutrophil infiltration, alveolar wall disruption and formation of hyaline membranes.

Cellular infiltration was estimated on the same regions of interests, using a cell counting procedure based on the software ImageJ (https://imagej.nih.gov/), targeting the cell nuclei stained by the hematoxylin. The number of nuclei counted was normalized to the tissue length present in each region of interest , obtaining a linear cell density. Tissue length was estimated with a custom made python script based on the package scikit-image^62^. Briefly the fraction of the total tissue surface present in the ROI associated to a given tissue thickness was estimated using morphological granulometry^63^, i.e. successive morphological opening with a kernel of increasing radius. Total tissue length was estimated accordingly, by dividing the tissue surface to the corresponding tissue thickness.

## Supplementary information

Supplementary Information.

Supplementary Video 1.

Supplementary Video 2.

## Data Availability

The datasets analyzed in the current study are available from the corresponding author on reasonable request.
