# Supplementary material for: Imaging atelectrauma in Ventilator-Induced Lung Injury using 4D X-ray microscopy
Source: Sci Rep. 2021 Feb 19;11:4236. doi: 10.1038/s41598-020-77300-x (PMC7895928; doi:10.1038/s41598-020-77300-x)
Supplement: Supplementary file 1 — Supplementary Information. [file 41598_2020_77300_MOESM1_ESM.docx]

**Supplemental Digital Content**

**Imaging atelectrauma in Ventilator-Induced Lung Injury using 4D X-ray microscopy**

Luca Fardin, PhD^1,2,7^, Ludovic Broche, PhD^1^, Goran Lovric, PhD^3,4^, Alberto Mittone, PhD^5^, Olivier Stephanov, MD^6^, Anders Larsson, MD-PhD^2^, Alberto Bravin, PhD^1,7^, Sam Bayat* MD-PhD^6,7^

**Supplemental Tables**

| *PaO_2_/F_IO2_ (mmHg)* | *Baseline* | *Injury* | *End* |
| --- | --- | --- | --- |
| Rabbit 1 | 384 | 100 | 84 |
| Rabbit 2 | 500 | 107 | 318 |
| Rabbit 3 | 508 | 66 | 50 |
| Rabbit 4 | 478 | 74 | 40 |
| Rabbit 5 | 434 | 41 | 43 |
| mean ± SD | 460 ± 50 | 80 ± 30* | 110 ± 120* |

**Table S1.**


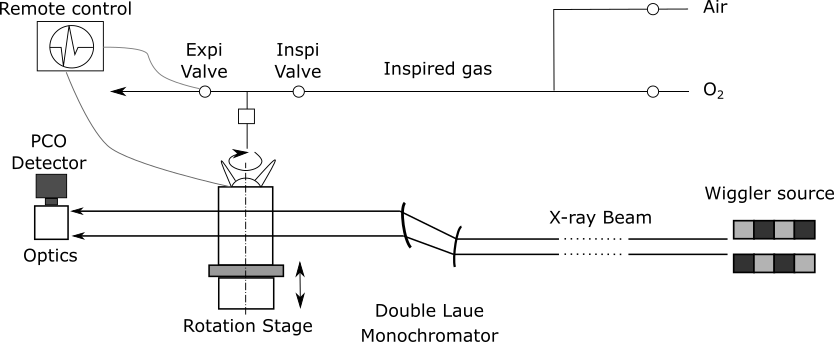


**Figure S1.**


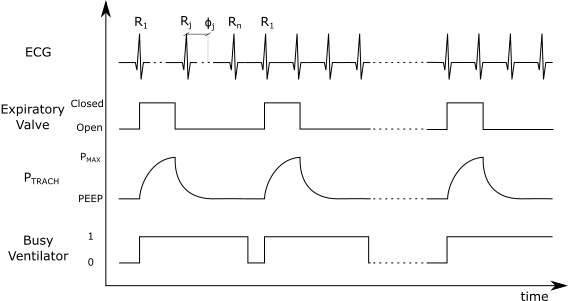


**Figure S2.**

**Supplemental Figure Legends**

**Table S1.** PaO2/FIO2 ratio at baseline, at the end of injurious ventilation and at the end of the experiment. *: p<0.05 vs. baseline.

**Figure S1.** Scheme of the experimental setup for in vivo lung imaging. The animal is immobilized in a plastic holder and placed on the rotation stage. The custom-made mechanical ventilator consists of: (i) an inspiratory branch connected to oxygen and air bottles, which allow to control the flow and the fraction of inspired oxygen; (ii) electromagnetic valves allowing remote control of breathing and synchronization with the heartbeat. For imaging, the animal is irradiated with monochromatic radiation and projections are acquired with a Complementary Metal Oxide Semiconductor (CMOS) detector.

**Figure S2.** Synchronization of the mechanical ventilation with the heartbeat. The Labchart software is used to monitor the ECG. When an R wave is detected, a square Transistor-Transistor logic (TTL) signal is generated, with a duration and duty cycle determined by the user and sent to the expiratory valve. A logical value of 1 (0) closes (opens) the expiratory valve, determining lung inflation (deflation). The polling on the ECG is disabled for the whole duration of the square wave, concept represented as the busy signal of the mechanical ventilator. The phase of the periodic parenchymal motion is determined by the delay Φj from the j-th R wave of the respiratory cycle.

**Video S1.** Example of in vivo lung dynamic 3D-microscopy reconstruction for Rabbit-1.

**Video S2.** Recruitment and derecruitment map as a function of time during a whole respiratory cycle for Rabbit-1. Each time frame represents a 2D projection of the 3D rendering of the recruitment (green) and derecruitment (red) as identified by our algorithm. Recruitment and derecruitment are computed comparing consecutive time frames and do not contain therefore cumulative information.
